# Crosslinking bacterial postbiotics for microbial and quality control of strawberries postharvest: bacteriological and 16S amplicon metagenome evidence

**DOI:** 10.3389/fmicb.2025.1570312

**Published:** 2025-03-19

**Authors:** Gabriela N. Tenea, Pamela Reyes, Carlos Flores

**Affiliations:** Biofood and Nutraceutics Research and Development Group, Faculty of Engineering in Agricultural and Environmental Sciences, Universidad Técnica del Norte, Ibarra, Ecuador

**Keywords:** postbiotics, antimicrobials, strawberries, *Serratia liquefaciens*, 16S amplicon

## Abstract

**Introduction:**

Strawberries are renowned for their exceptional flavor and nutritional properties but have a short shelf life due to rapid ripening and a high vulnerability to postharvest microbial decay. Postbiotic formulations (PBFs) derived from lactic acid bacteria (LAB) can be developed into effective preservation products, extending postharvest shelf life while maintaining fruit quality.

**Methods:**

This study aimed to assess the effects of postbiotic-based formulations (PBFs) consisting of two key components: (1) a precipitated peptide-protein extract (PP) from *Weissella cibaria* UTNGt21O, serving as the antimicrobial agent, and (2) an exopolysaccharide (EPS) from *W. confusa* UTNCys2-2, functioning as the biopolymer carrier. These formulations were tested against a multidrug-resistant *Serratia liquefaciens* P4StpC1 strain, isolated from ready-to-eat strawberries, and their potential mode of action was analyzed *in vitro*. Time-kill assays and electron microscopy were used to evaluate their impact on the target cells. Furthermore, the performance of PBFs was compared to a commercial disinfectant (C1) in terms of their effects on strawberry microbiota and fruit quality, employing bacteriological techniques and 16S amplicon metagenomic analysis.

**Results:**

The selected PBFs showed bacteriolytic effect on *Serratia in vitro*. The target cell viability was significantly reduced upon 1 h co-cultivation by inducing several morphological and ultrastructural modifications. Dipping strawberries at the ripe stage four in PBFs indicated no increase in total cell counts, thus the microorganisms colonization was retained during storage with refrigeration. The 16S metagenome analysis showed that the treatment impacted the fruit microbiota, significantly increasing *Lactobacillus* abundance (*p* < 0.001) by day eight compared to the disinfectant control. This suggests the formulation supports beneficial microbes, enhancing antimicrobial effects. Additionally, the postbiotic coating improved shelf-life, preserved fruit quality, and delayed deterioration in strawberries. The strawberries quality attributes were not affected by the treatment. Principal Component Analysis (PCA) revealed clear sample separation based on maturity stage, independent of the treatment.

**Conclusion:**

The results highlight the potential of crosslinking of a peptide-protein fraction with EPS to prevent the colonization of undesirable microorganisms on postharvest strawberries while enhancing their safety and quality.

## 1 Introduction

Various bacteria and fungi can adhere to fruits, especially those consumed with their exocarp (e.g., strawberries, apples, blackberries, pears), during both pre- and postharvest stages, posing risks to fruit safety and quality (Founou et al., 2021). Several studies have shown that these microorganisms can act as “incubators” for antibiotic-resistant genes (ARGs) (Rahman et al., 2021). Common ARG carriers found on fruits, vegetables, and sprouts include *Bacillus cereus*, *Enterobacter* spp., *Listeria* spp. (mainly *L. monocytogenes*), *Rahnella aquatilis*, *Staphylococcus* spp., *Shigella* spp., and *Citrobacter* spp. (Rahman et al., 2021).

Strawberries are a popular fruit worldwide, valued for their health benefits and exceptional flavor. However, they are highly prone to postharvest spoilage due to factors like nutrient oxidation and moisture loss (Priyadarshi et al., 2024). Recent studies have identified *Serratia liquefaciens*, an opportunistic pathogen from the Enterobacteriaceae family, as a key contributor to diseases in strawberry cultivars such as ‘Mara des Bois’ and ‘White Ananas’ (Olimi et al., 2022). Through amplicon metagenomics targeting the 16S rRNA and ITS2 regions, we have revealed that strawberries host a diverse range of bacteria and fungi (Tenea and Reyes, 2024; Tenea et al., 2024a). Market-originating strawberries showed a high prevalence of *Serratia* spp. (>60%), while field samples were abundant in *Pseudomonas* spp. (>70%). Both market and field samples contained *Salmonella enterica* and *Escherichia coli*, but only market samples harbored *Enterococcus gallinarum*, *Shewanella putrefaciens*, and *S. profunda* (Tenea and Reyes, 2024). Additionally, market fruits contained more potentially harmful fungi, including the clinically significant yeasts *Candida parapsilosis* and *Meyerozyma guilliermondii* (Tenea et al., 2024a).

To combat microbial contamination and extend strawberry shelf life, eco-friendly methods, particularly biopolymer-based (polysaccharides, lipids, and proteins) edible coatings have been applied (Priyadarshi et al., 2024). The edible film is a thin layer of material that functions as a semi-permeable physical barrier to prevent mechanical damage and deterioration to fruit skin, delay dryness, slow decline, and regulate gas (CO2/O2) exchange (Sogvar et al., 2016). In addition, cassava starch–based bionanocomposite coatings preserved postharvest strawberry quality by slowing senescence and inhibiting fungal growth, offering a biodegradable alternative to conventional packaging (Carvalho do Lago et al., 2023). Although these methods are inexpensive, and environmentally friendly, practical application is limited due to challenges in scaling up production (Priyadarshi and Rhim, 2020). On the other hand, these materials should be organic, non-toxic and chemical-free (Ali et al., 2022). However, to preserve the quality of strawberries a recent developed method combines the laser irradiation with a coating of chitosan nanoparticles derived from guava leaves (Ali et al., 2022).

We recently developed antimicrobial formulations using metabolites from lactic acid bacteria (LAB) that successfully protected immature avocados from bacterial colonization (Tenea et al., 2024b). Among the tested LAB strains, *Weissella cibaria* UTNGt21O and *W. confusa* UTNCys2-2 showed strong antimicrobial activity, making them promising candidates (Tenea and Hurtado, 2021). This study aims to evaluate the antimicrobial efficacy and mode of action of a postbiotic-based formulation (PBFs) combining a peptide-protein extract from *W. cibaria* UTNGt21O as an antimicrobial agent and an exopolysaccharide from *W. confusa* UTNCys2-2 as a biopolymer carrier. The PBFs was tested at varying doses against a multidrug-resistant *Serratia liquefaciens* strain P4StpC1, isolated from ready-to-eat strawberries. Time-kill assays and electron microscopy were employed to assess its impact on bacterial cells. Additionally, the study investigates the PBFs ability to prevent microbial colonization on postharvest strawberries compared to a commercial disinfectant, using bacteriological methods and 16S amplicon metagenomics. Furthermore, the effects of PBF on the strawberries functional properties (total polyphenol content, antioxidant capacity, ascorbic acid content) and physicochemical characteristics (pH, total soluble solids, titratable acidity) were analyzed.

## 2 Materials and methods

### 2.1 Bacterial strains, PBFs composition, and antimicrobial activity against *Serratia liquefaciens* P4StpC1 *in vitro*

*Weissella cibaria* UTNGt21O (GenBank Genome Assembly SRX8614718) and *W. confusa* UTNCys2-2 (GenBank accession number KY041684.1) were previously isolated from tropical fruits (Tenea and Hurtado, 2021). Fresh cultures were prepared by cultivating the strains on MRS (Man, Rogosa, and Sharpe) agar (Difco, USA) at 37°C prior to use. The multidrug resistant strain *Serratia liquefaciens* P4StpC1, isolated from strawberries, was cultured in BHI (Brain Heart Infusion, Merck Millipore, MA, USA) broth medium. All microorganisms were stored at −80°C in 20% glycerol (v/v).

Cell-free supernatant (CFS) from an overnight culture of UTNGt21O grown in MRS broth at 37°C was obtained as described by Garzón et al. (2017). The filtered CFS was precipitated using ethyl acetate (v/v), incubated for 24 h at low temperature without stirring, and centrifuged at 8000 × g for 30 min. The resulting precipitated peptides-proteins (PPGt21O) were resuspended in 25 mM ammonium acetate (pH 6.5), desalted using a midi dialysis kit (#PURD10005-1KT, Sigma-Aldrich Co. LLC, Saint Louis, MO, USA) pre-equilibrated with phosphate buffer (pH 7.0), dried under a flow chamber for 48 h, reconstituted in sterile water, and stored at −20°C. For exopolysaccharide (EPS) extraction from UTNCys2-2, an overnight bacterial culture (1 × 10^8^ CFU/mL) was grown in MRS broth supplemented with 20% sucrose (MRSS). The EPS extraction protocol followed previously established methods (Wang et al., 2023). The antimicrobial activity of the formulations was tested using the agar-well diffusion method. A detailed description of the formulations and their concentrations is provided in Supplementary Table S1. The experiment was initiated with independent cultures and performed in triplicate.

### 2.2 Minimum inhibitory concentration (MIC) determination and co-culture of selected PBFs with P4StpC1 cells

The MIC was determined following the method described by Xiang et al. (2021). Briefly, a two-fold serial dilution of the formulation was prepared and added to the indicator bacteria. After an 18-h incubation at 37°C, the PBFs were tested at varying concentrations ranging from 800 to 12,800 AU/mL. Viable bacterial counts were assessed using the plate count method, and the MIC was calculated as the concentration that reduced the target bacterial growth by 50% compared to untreated controls (Arena et al., 2016). The MIC (1×) was determined to be 1,600 AU/mL. To assess the time-dependent effect of PBFs, a time-kill co-culture experiment was conducted as described by Wang et al. (2022). An overnight culture of *S. liquefaciens* P4StpC1 (1 × 10^6^ CFU/mL) was inoculated with PBFs at a concentration equivalent to 1× MIC and incubated at 37°C. Untreated cells served as the control. Cell viability was measured at various time intervals (0, 1, 2, 3, and 4 h) using the plate count agar method (BD Difco plate count agar, Fisher Scientific Co. LLC, Hampton, NH, USA). The control consisted of broth medium without any formulation added. Each sample was tested in triplicate, and the experiments were performed independently three times.

### 2.3 The ultrastructural and morphological changes examination of P4StpC1 upon PBFs treatment

P4StpC1 cells at the exponential phase (1 × 10^6^ CFU/mL) were treated with 1× MIC of selected PBFs for 6 h at 37°C. Using a Tecnai G2 F20 transmission electron microscope (FEI Company, Hillsboro, OR, USA), 10 randomly chosen sections from each treatment were examined. The samples were placed on graphite tape for SEM analysis. A thin layer of gold, approximately 24.5 nm thick, was applied to each sample using DENTON VACUUM Desk IV equipment (DENTON VACUUM, Austin, TX, USA). The samples were then examined with a high vacuum scanning electron microscope to produce high-resolution images. The morphology and topography of the samples were examined using the secondary electron detector.

### 2.4 Effect of selected PBFs on strawberry fruits during storage

#### 2.4.1 Application of PBS to strawberries *in vitro*

The strawberries (64 samples, 25 fruits × 4 repetitions × 4 treatments × 4 time-interval = 1,600 fruits) were purchased from a local producer (Otavalo, city) during February to October 2024. The fruits were selected at ripeness stage four (more than 50% red) and had no visible damage. They were rinsed with a 5% bleach solution for 5 min, then twice with tap and twice with distillate water, and allowed to dry for approximately 2 h under a biosafety cabinet. The fruits were dipped in T1 (1 × MIC, PPGt21O + EPSCys2-2: 1:1, v/v), T2 (PPGt21O, 1 × MIC), T3 (EPSCys2-2, 1 × MIC) solutions, C1, a commercial disinfectant (Star Bac Domestic, a bactericidal solution) made in accordance with the manufacturer’s instructions for 10 min in a final volume of 300 mL. The fruits were let dry and transferred in plastic trays and stored for 8 days with refrigeration. A control group C (total 16 samples), of water washed fruits was included (25 fruits × 4 repetition × 4 time-interval, total 400 fruits).

#### 2.4.2 Bacteriological analysis: culture-dependent assay

Bacteriological analyses were conducted on days 0, 1, 4, and 8, following the method described by Angamarca et al. (2023). In summary, each treatment consisted of 25 fruits that were individually placed in a Ziplock bag with distilled water at room temperature for an hour. The microbial cells were collected by centrifugation at 8000 × g for 5 min and resuspended in 10 mL of 1× PBS. The presence of target microorganisms was evaluated using specific agar media: Chromocult Coliform agar (Merck Millipore, Kenilworth, NJ, USA) to assess total coliforms and the potential presence of *Escherichia coli* (TCOL); eosin methylene blue agar (Difco, Detroit, MI, USA) to detect both *Enterobacter* spp. (ENT) and *E. coli*; and Dichloran Rose-Bengal Chloramphenicol (DRBC) Agar Base (Thermo Scientific™ Oxoid™, USA) for the detection and enumeration of molds (M) and yeasts (YE) after incubation for 7 days at 25–28°C. All experiments were performed in triplicate, and microbial counts were expressed as CFU/g. Additionally, fruit visual changes, including color and appearance (e.g., the development of brownish spots or fungal growth), were monitored and recorded for both treated and untreated (water-washed) samples over the 8-day storage period.

#### 2.4.3 16S rRNA gene metagenomics: culture-independent assay

DNA extraction, Library Construction, Sequencing, data processing and analysis.

A total of 32 samples (25 fruits × 4 repetitions × 4 treatments × 2 time intervals) were used in the study. To collect the cells surrounding the fruit skin (exocarp), the strawberries treated with PBFs and the commercial control underwent centrifugation at 8,000 × g for 5 min. Samples were collected at two time points: the initial stage (1 day after treatment) and the final storage stage (day 8). The recovered cells were suspended in 1× PBS and stored at 4°C prior DNA extraction. Sequencing, library building, DNA extraction, and quantification were carried out as previously mentioned (Tenea and Reyes, 2024). In summary, a quality and filtering procedure was applied to FASTq files to ensure taxonomic classification. Marker gene-based microbiome sequencing data analysis was performed using the QIIME v.2 (Quantitative Insights into Microbial Ecology) pipeline (Bolyen et al., 2019), version 2023.5. The 16S region was extracted after the sequences were denoised using the denoise wrapper (Reeder and Knight, 2010). Moreover, DADA2 was used to group sequences (clustering) and remove noise (denoising) (Callahan et al., 2020). Higher-resolution tables of amplicon sequence variables (ASVs) are produced by DADA2 (Alishum, 2021). USEARCH 6.1 was used to find and filter chimeric sequences (Edgar, 2010). Furthermore, the sequences are given a taxonomy. Additionally, a reference database of sequences with known taxonomic composition was compared to the query sequences (ASVs). The method involves applying a multinomial naive Bayes classifier using machine learning to train a classifier on a reference database and then using the trained classifier to classify ASVs. This classifier is trained with the target region of interest V3-V4 based on the SILVA reference taxonomy version 138, obtained from Zenodo (Mohsen, 2021). Sequences belonging to chloroplasts, mitochondria, and eukaryotes were removed.

#### 2.4.4 Rarefaction curves, alpha and beta diversity analysis, and significance tests

According to Kandlikar et al. (2018), rarefaction curves are helpful in determining how sequencing depth impacts the capacity to fully capture a sample’s diversity. The metrics listed below were established for alpha diversity: Observed features, which is a qualitative measure of community wealth; Faith’s phylogenetic diversity, which considers the phylogenetic relationships between traits; the Shannon diversity index, which measures species diversity within groups; and evenness/Pielou uniformity, which measures variety in addition to species richness (Krebs, 2014). Alpha diversity metrics were compared using either a nonparametric Kruskal-Wallis test or a one-way ANOVA on ranks to assess whether the samples originated from the same distribution. Beta diversity was evaluated using two approaches: non-phylogenetic (Bray–Curtis dissimilarity) and phylogenetic (UniFrac distance) metrics, as described by Lozupone et al. (2007). Statistical significance of differences in beta diversity was determined using 999 Monte Carlo permutations, with weighted and unweighted UniFrac distance matrices. Principal coordinates analysis (PCoA) in QIIME 2.0 was employed to link the bacterial community composition to sample groups, and relationships in beta diversity were visualized as two- or three-dimensional scatter plots. A significance threshold of 0.05 was applied for all statistical tests. Moreover, the analysis of similarities (ANOSIM) was used to evaluate the hypothesis that no differences exist between two or more sample groups, based on permutation testing of within- and between-group similarities. The R test statistic was used to determine the validity of the null hypothesis (Yinglin et al., 2018). Additionally, taxa with significantly different abundances across groups were identified using ANCOM-BC (Analysis of Composition of Microbiomes with Bias Correction) (Lin and Peddada, 2020). The resulting analysis provided a graphical representation of differences in taxonomic abundance between the groups of interest and the reference group.

### 2.5 Fruit quality evaluation during storage

Fruit quality was assessed as described (Tenea et al., 2024b). pH, total titratable acidity (TTA), total soluble solids (TSS), total polyphenol content (TPC) estimation, ascorbic acid content (AAC), and antioxidant (AOX) activity were determined in all treated (64) and untreated (16) samples during storage (days 0, 1, 4, and 8). An electrode immersion pH meter (S210, Mettler Toledo, Columbus, OH, USA) was used to measure the pH. 25 mL of fruit juice was titrated with 0.1 N NaOH to determine the TTA using phenolphthalein as an indicator (AOAC, 2003). The percentage of ascorbic acid in 100 milliliters of juice was used to express the results. A digital refractometer was used to measure the TSS (AOAC, 2003). The Folin–Ciocalteu method with gallic acid (Sigma-Aldrich Co. LLC, Saint Louis, MO, USA) was used to determine TPC during storage. The TPC result was expressed as milligrams of gallic acid equivalents (GAE) per gram (mg GAE/g) of the sample, and the calibration curve was generated using a gallic acid standard (0–200 μg/mL). The DPPH (1, 1-diphenyl-2-picryl-hydrazyl, Sigma-Aldrich Co. LLC, Saint Louis, MO, USA) radical scavenging activity was assessed during storage (Ali et al., 2022). A Trolox standard was used to compare the capacity of AOX in a mixture sample to scavenge DPPH (Sigma-Aldrich Co., LLC, Saint Louis, MO, USA). A conventional 2,6-dichloroindophenol titrimetric method was used to measure the amount of vitamin C (reduced ascorbic acid) (AAC) (AOAC, 2003). Beginning with a fresh batch of fruit extracts, the analyses were performed in triplicate. In addition, the fruit firmness (Newtons, N), a cylindrical probe with a 5 mm diameter was used to puncture the fruit’s equatorial side (Ren et al., 2023). A compact universal/tensile benchtop tester (Shimadzu Scientific Instruments, Japan) was used, and data was analyzed using Trapezium X software. A whole fruit was placed lengthwise on the measuring plate, and the probe was inserted through the skin into the center of the fruit. The response factors were the mean work (Joules) and the load (Newtons) required to penetrate the skin were registered during storage. All fruits were similar in size and the measurements were done at the room temperature.

### 2.6 Statistical analysis

The mean ± standard deviation was used to present the results. Tukey’s *post hoc* test and the Kruskal-Wallis one-way analysis of variance (non-parametric) were employed to identify significant differences between the means (SPSS version 10.0.6, US). The cutoff value for statistical significance was set at *p* < 0.05 (SPSS version 10.0.6, US). Furthermore, a PCA of six variables (pH, TSS, TTA, AOX, TPC, and AAC) was performed on both treated and untreated fruits (Tenea et al., 2024b). Pearson correlation was used to ascertain whether there was any interaction between the response variables.

## 3 Results and discussion

### 3.1 PBFs suppress *Serratia liquefaciens* P4StpC1 growth inducing morphological and ultrastructural cell changes

**Figure 1:**
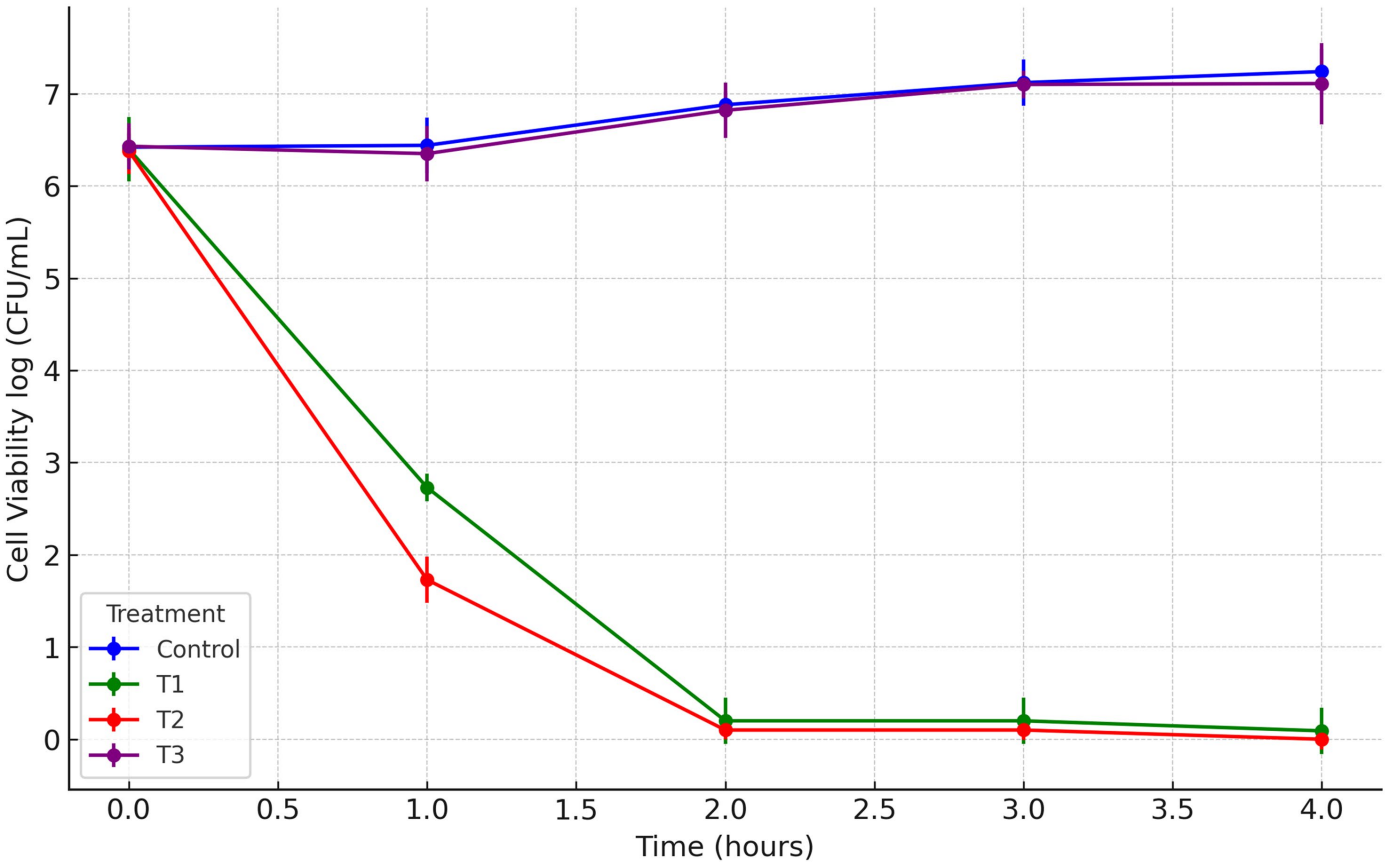
Time-kill curves of *Serratia liquefaciens* UTNP4StpC1. Dara represents the cell viability reported as logCFU/mL. Error bars represent the standard deviations of three replicates (*n* = 3). T1: (1 × MIC) PPGt21O + EPSCys2-2 (1:1, v/v); T2: (1 × MIC) PPGt21O; T3: (1 × MIC) EPSCys2-2; Control: P4StpC1 untreated cell culture.

**Figure 2:**
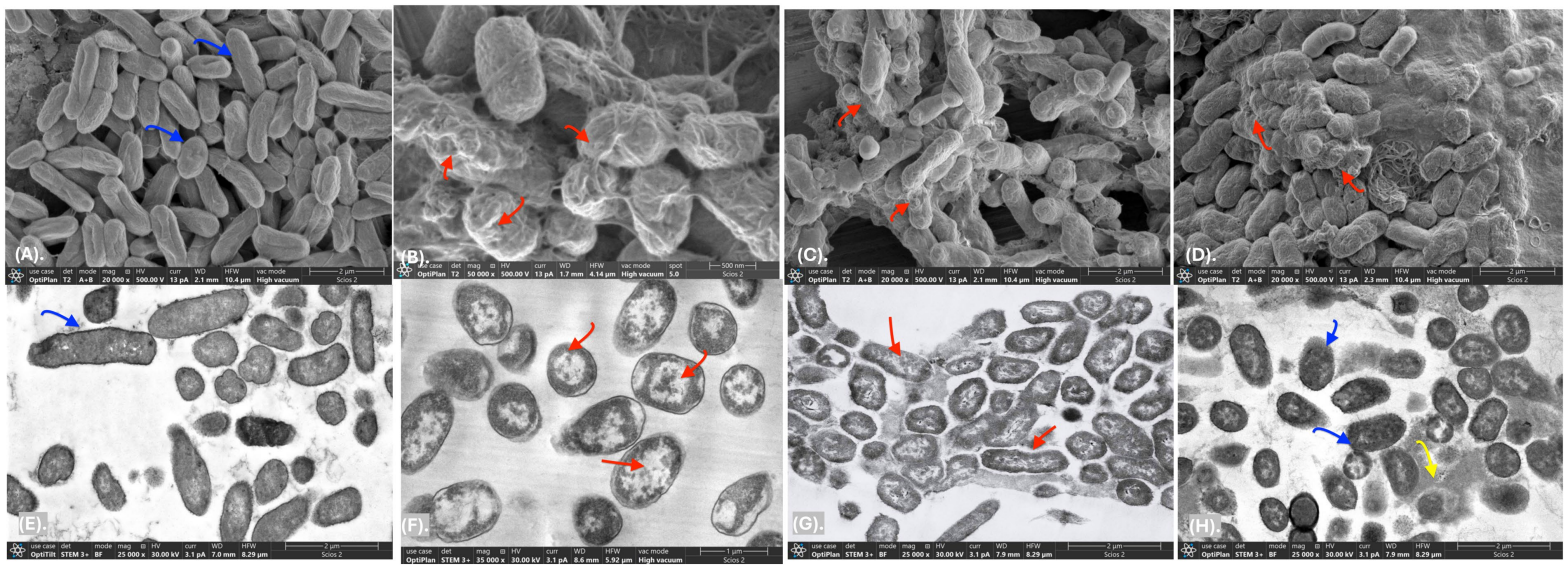
SEM and TEM of *Serratia liquefaciens* P4StpC1. **(A–E)** control untreated cells; **(B–F)** cells treated with T1 1 × MIC (PPGt21O + EPSCys2-2, 1;1, v/v); **(C–G)** cells treated with T2 (PPGt21O, 1 × MIC); **(D–H)** cells treated with T3 (EPSCys2-2, 1 × MIC) at 1 × MIC, 6 h. Scale bars correspond to 500 nm, 1–2 μm. Blue arrows: rod-like shapes with intact membranes and nucleoid structures; Red arrows: membrane damage, shape deformation, and cell entrapment, DNA relaxation, and filament formation; Yellow arrows: EPS layer.

In this study, a postbiotic-based formulation (PBF) was developed to target the multidrug-resistant *S. liquefaciens* strain P4StpC1. Previous genome analysis revealed that the antimicrobial activity of *W. cibaria* UTNGt21O is linked to the presence of the *enterolysin A* gene (Tenea and Hurtado, 2021). Meanwhile, *W. confusa* UTNCys2-2 produces exopolysaccharides (EPS) from sugar-containing media, which are recognized as biodegradable biopolymers and potential food thickeners (Prete et al., 2021). The viscous nature of EPS was hypothesized to enhance the delivery of the antimicrobial fraction to fruit surfaces. To investigate this, the antimicrobial fraction was crosslinked with EPS, and the activity of the resulting formulation was evaluated *in vitro*. Agar well-diffusion assays revealed that treatments T1 (PPGt21O + EPSCys2-2) and T2 (PPGt21O) demonstrated the strongest inhibitory activity, while T3 (EPSCys2-2) showed minimal effects (Supplementary Table S1). In co-culture assays with *S. liquefaciens* P4StpC1, T1 and T2 significantly reduced viable cells within 1 h (98.4 and 99%, respectively), with no detectable viable cells after 2 h (Figure 1), indicating a bacteriolytic effect primarily driven by the PPGt21O fraction. Conversely, T3 showed no inhibitory activity, and cell viability increased, confirming that EPS alone does not inhibit the target pathogen. These findings are consistent with prior research demonstrating the bacteriolytic activity of PPGt21O against methicillin-resistant *Staphylococcus* strains isolated from avocados (Tenea et al., 2024b). To further investigate the mechanism of action, scanning and transmission electron microscopy (SEM and TEM) analyses were conducted. Untreated *Serratia* cells showed smooth, rod-like shapes with intact membranes and nucleoid structures (Figures 2A,E). In contrast, cells treated with T1 and T2 exhibited significant alterations, including membrane damage, shape deformation, and cell entrapment, suggesting direct interactions between the PBF components and the bacterial surface (Figures 2B–D). These results indicate that the bacterial cell membrane is likely the primary target, with the disruption of membrane integrity ultimately leading to cell death. TEM provided additional insights, revealing morphological changes such as separation of the cell envelope, DNA relaxation, and filament formation, further confirming damage to membrane integrity and cellular function (Figures 2F,G). No such effects were observed with T3, where the cells remained intact and surrounded by the EPS layer (Figure 2H). These morphological changes are reminiscent of those seen in *Enterobacteriaceae* treated with antibiotics like colistin and imipenem (Hisada et al., 2023). The results suggest that the PBF disrupts bacterial membranes, compromising their structural integrity and resulting in cell death. This aligns with previous studies showing that PPGt21O disrupts the membranes of *E. coli*, *Salmonella* spp., and *Staphylococcus aureus*, demonstrating its broad-spectrum antibacterial activity (Tenea and Hurtado, 2021). Future research will focus on further chemical characterization of the formulations to deepen our understanding of their inhibitory mechanisms.

### 3.2 PBFs impairs microorganisms colonization on strawberry skin during storage

**Figure 3:**
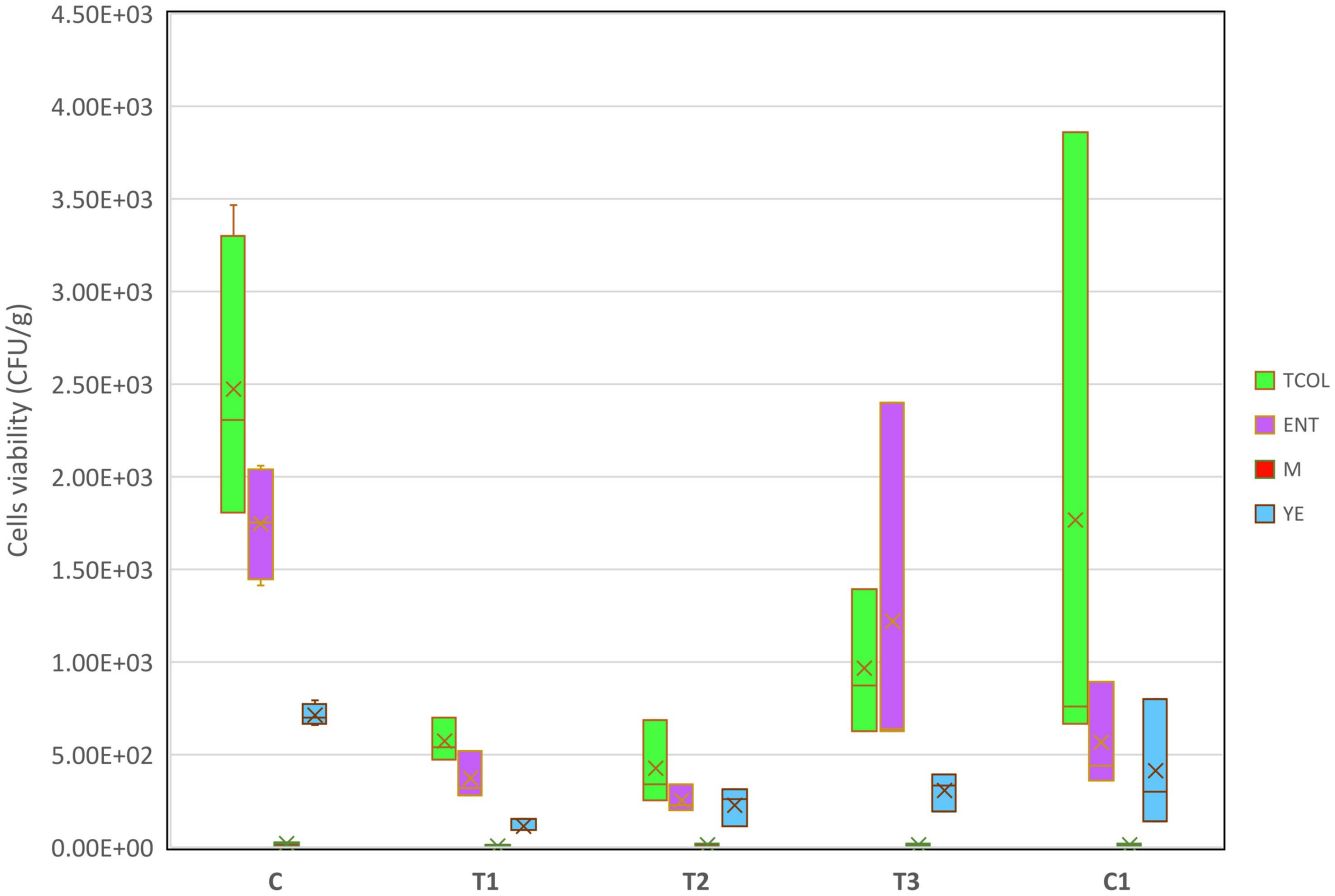
Boxplots of target microorganisms upon the treatment with PBFs. Each box’s middle line aligns with the mean, and its upper and lower bounds are spaced one standard deviation apart from the mean. The highest and lowest values of each group are reached by the whiskers that extend above and below the box. The significance was determined Kruskal-Wallis; the values were considered significant when *p* < 0.05. T1: (1 × MIC) PPGt21O + EPSCys2-2 (1:1, v/v); T2: (1 × MIC) PPGt21O; T3: (1 × MIC) EPSCys2-2; C, P4StpC1 untreated cell culture; C1, commercial disinfectant treated cells; TCOL, total coliforms; ENT, Enterobacter spp., M, molds; YE, yeasts.

To prevent microbial colonization during storage, postbiotic formulations can be applied as transparent biofilms on fruit surface (Tenea et al., 2024b). However, their efficacy may vary depending on the specific fruit, as exocarp composition and microbiota differ. In this study, we hypothesized that fruit deterioration during storage could be mitigated, or quality enhanced, by treating strawberries with a polymer carrier containing an active antimicrobial metabolite. Ripe, undamaged, and fungus-free strawberries at stage four maturity were selected (Supplementary Figure S1A) and treated with PBFs or a commercial disinfectant (C1), and their phenotypic characteristics—such as color, texture, and fungal growth—were monitored over 8 days of storage (Supplementary Figure S1B). Control fruits (C, water-washed) exhibited wrinkling, opacity, and significant water loss, rendering them unfit for consumption. Fruits treated with C1 became opaque and dry. In contrast, those treated with T1 (PPGt21O + EPSCys2-2) and T2 (PPGt21O) retained smooth surfaces and vibrant red color, though their texture softened slightly. Fruits treated with T3 (EPSCys2-2) developed a high gloss, likely attributed to the polymeric properties of the EPS. Although the amount of active ingredient absorbed by the fruit exocarp remains undetermined, T3 alone appeared to accelerate fruit degradation. By day eight, statistically significant differences (*p* < 0.05) in microbial counts were observed between groups. Control (C) and C1-treated fruits exhibited high levels of *Enterobacter* spp., total coliforms (TCOL), and yeasts (YE). Conversely, T1 and T2 treatments effectively inhibited microbial colonization, showing no significant increases in cell counts during storage (Figure 3). Mold (M) growth remained stable across all treatments, suggesting the formulations effectively suppressed fungal proliferation. These findings are consistent with earlier research on avocados, where active molecules in postbiotic formulations served as protective barriers, reducing microbial growth (Tenea et al., 2024b). Recent research explored the use of active adsorbent pads made from cellulose infused with chitosan or limonene to preserve strawberries inoculated with *Botrytis* (Cefola et al., 2023). The activated pads delayed microbial growth and maintained the strawberries’ fresh appearance for an additional 3 days. The inclusion of limonene helped retain the fruit’s freshness by preserving its volatile components, while chitosan activation significantly reduced the fruit’s respiratory activity (Cefola et al., 2023). However, despite delaying microbial growth and lowering respiratory activity, the pads showed limited antifungal efficacy, as chitosan did not significantly inhibit fungal proliferation. In the current study, PBFs effectively suppressed both bacterial and fungal growth during storage. These results indicate that PBFs provide a stronger and more effective approach to managing microbial growth. Future studies should focus on identifying the active metabolites in these formulations to determine whether their bacteriolytic effects stem from individual compounds or synergistic interactions. This study underscores the promise sustainable and effective alternative of PBFs to chemical disinfectants for fruit preservation. Such approaches meet consumer demand for eco-friendly preservation methods while addressing key challenges in fruit storage and transportation.

### 3.3 Microbial diversity and composition in strawberries upon the treatment with the PBFs

**Figure 4:**
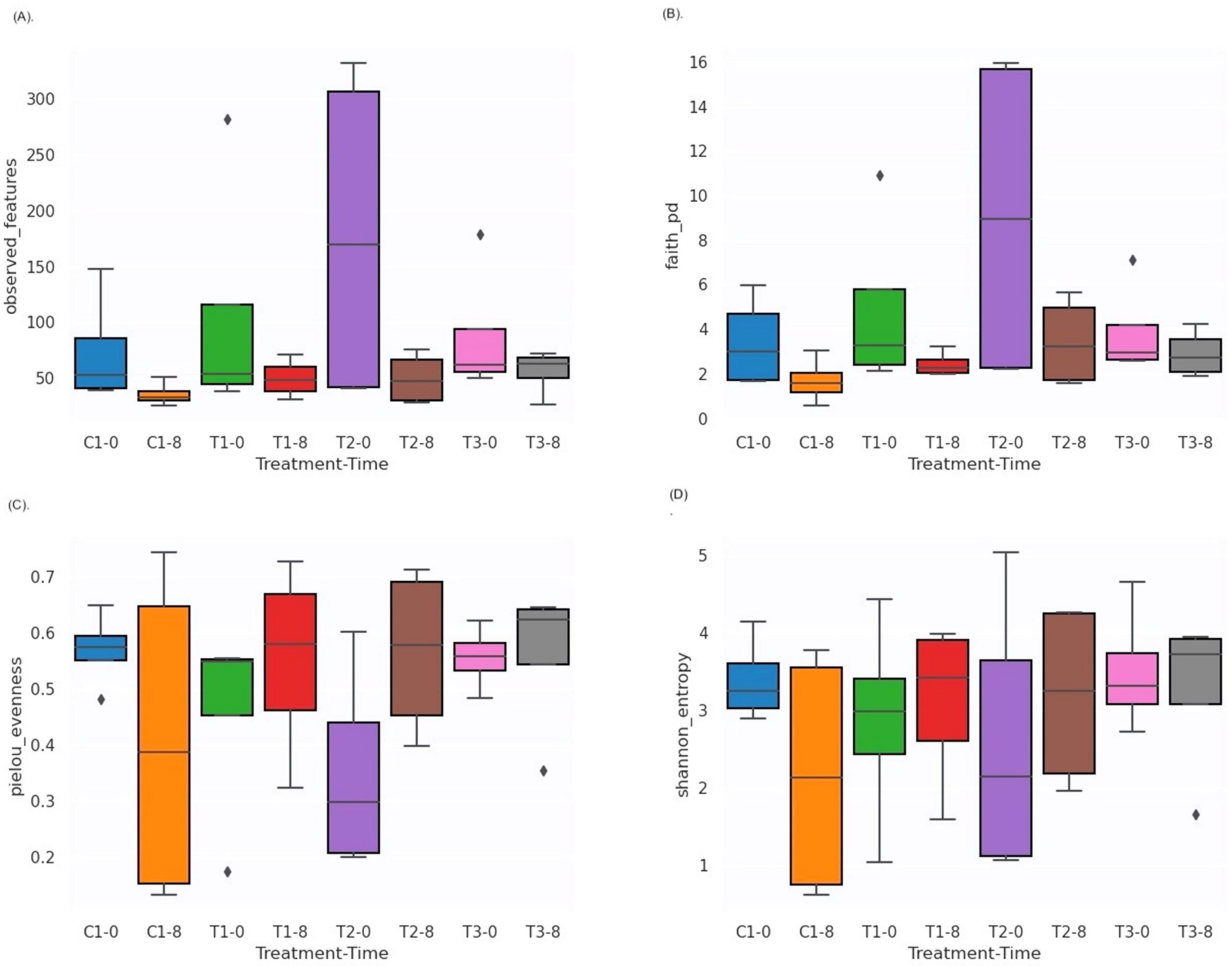
Box and whisker plots of alpha diversity values of analyzed bacterial communities to compare the Observed features **(A)**; Faith’s PD **(B)**; Shannon diversity index **(C)**; Pielou’s evenness **(D)**. C1-0, C1-8: fruits treated with commercial disinfectant, time initial (0) and final (8); T1-0, T1-8: treatment with 1 × MIC (PPGt21O + EPSCys2-2, 1:1 v/v) time initial (0) and final (8); T2-0; T2-8: treatment with PPGt21O (1 × MIC) time initial (0) and final (8); T3-0, T3-8: treatment with EPSCys-2-2 (1 × MIC) time initial (0) and final (8).

**Figure 5:**
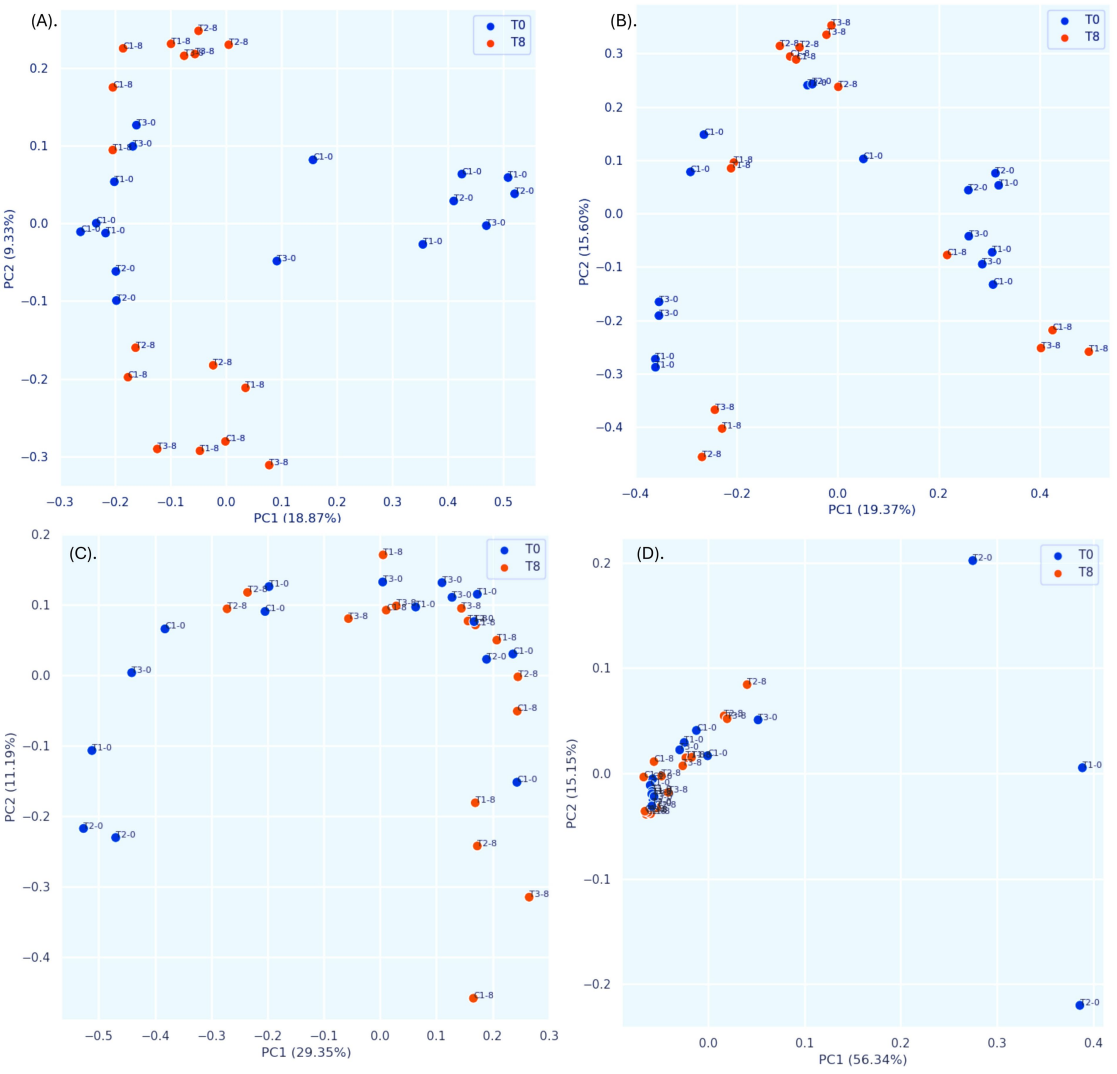
Principal Coordinate Analysis (PCoA) plots of bacterial beta diversity. **(A)** Jaccard distance; **(B)** Bray–Curtis dissimilarity indices; **(C)** unweighted UniFrac distance, **(D)** weighted UniFrac distance. Statistics were calculated using pairwise PERMANOVA with 999 permutations. C1-0, C1-8: fruits treated with commercial disinfectant, time initial (0) and final (8); T1-0, T1-8: treatment with 1 × MIC (PPGt21O + EPSCys2-2, 1:1 v/v) time initial (0) and final (8); T2-0; T2-8: treatment with PPGt21O (1 × MIC) time initial (0) and final (8); T3-0, T3-8: treatment with EPSCys-2-2 (1 × MIC) time initial (0) and final (8).

Unlike traditional bacteriological analysis targeting specific microorganisms, we used 16S amplicon metagenomics to analyze bacterial community composition and its changes in response to PBF and commercial disinfectant (C1) treatments. Samples were assessed on day 0 and day 8 of storage to evaluate microbial diversity and interactions. The characteristics of the input non-chimeric reads for each sample are summarized in Supplementary Table S2. No bacterial sequences were detected in the negative control. Rarefaction analysis demonstrated that a sequencing depth of 175,000 reads was sufficient to capture most taxa present in the samples, as indicated by the leveling of the rarefaction curve (Supplementary Figure S2). Alpha diversity, which reflects the distribution of microbes within a sample, including richness (number of taxa) and evenness (uniformity), showed no statistically significant differences between treatments or time points (days 0 and 8). This was evident from the Shannon index, observed features, evenness, and Faith’s phylogenetic diversity (Kruskal-Wallis test, Figures 4A–D, Supplementary Table S3). These findings are consistent with earlier research on nectarines treated with essential oils, which also reported no differences in alpha diversity but noted variability across different fruit parts (Remolif et al., 2024). In contrast, beta diversity analysis revealed significant differences between time points (T0 vs. T8) based on the Jaccard distance metric (pseudo-*F* = 1.812, *p* = 0.02; PERMANOVA, Figure 5A, Supplementary Table S4A). However, no significant differences were observed between treatment groups using the Bray–Curtis index (Figure 5B, Supplementary Table S4B). Phylogenetic analysis with the unweighted UniFrac distance metric showed significant differences between C1-T0 and C1-T8 (Figure 5C, Supplementary Table S4C), while no significant variation was detected using the weighted UniFrac distance (Figure 5D, Supplementary Table S4D). Additionally, ANOSIM analysis revealed minimal group separation, with R values close to zero, indicating a uniform microbial distribution across samples.

**Figure 6:**
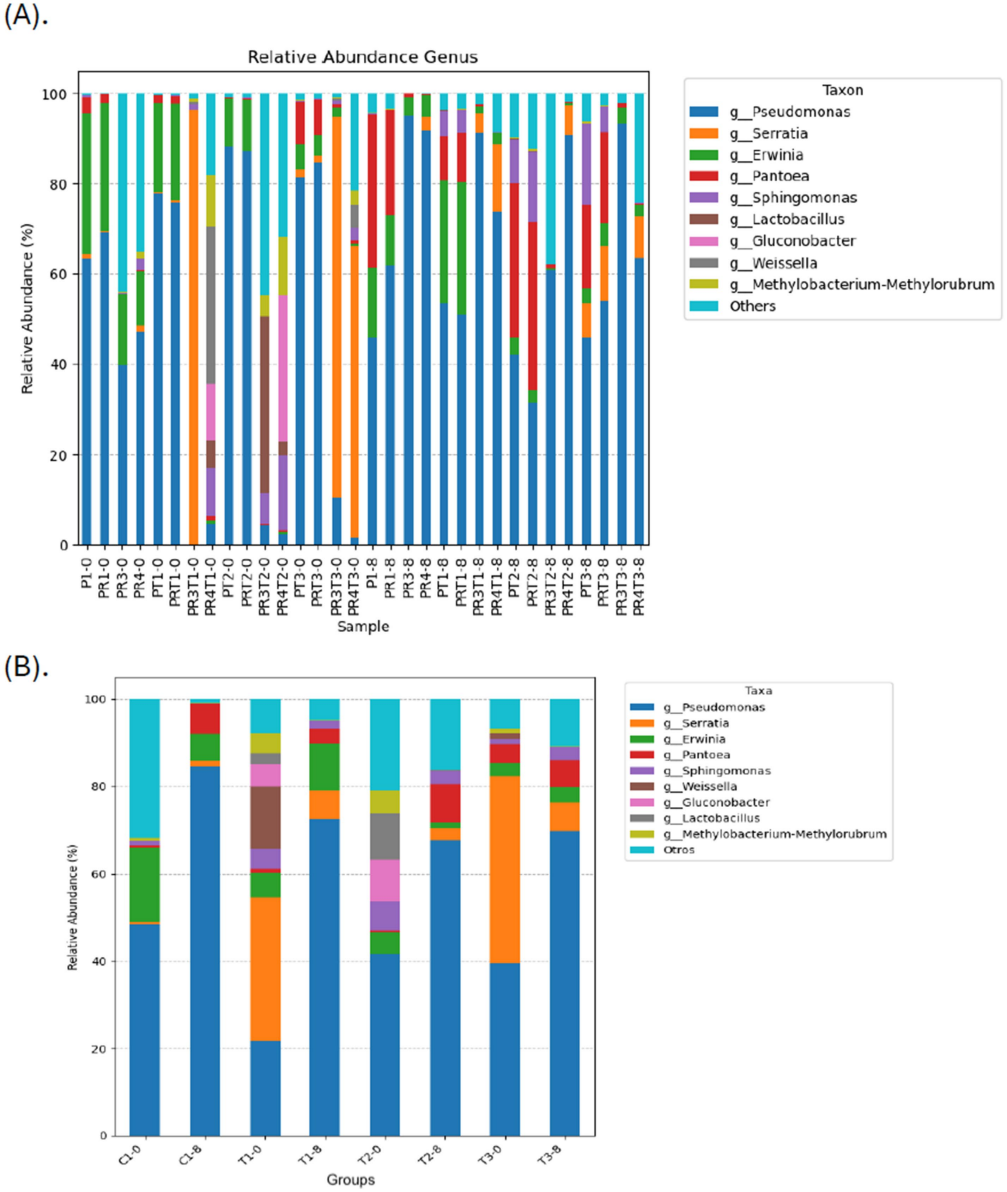
Relative abundance of different bacterial genera across the samples **(A)**, and among the groups **(B)**. This bar chart shows the relative abundance of the top 10 classification results within each taxonomic level. The “Other” category in this sum of all classifications with less than 0.50% abundance. C1-0, C1-8: fruits treated with commercial disinfectant, time initial (0) and final (8); T1-0, T1-8: treatment with 1 × MIC (PPGt21O + EPSCys2-2, 1:1 v/v) time initial (0) and final (8); T2-0; T2-8: treatment with PPGt21O (1 × MIC) time initial (0) and final (8); T3-0, T3-8: treatment with EPSCys-2-2 (1 × MIC) time initial (0) and final (8). The samples ID are detailed in Supplementary Table S2.

Similarly, a recent study evaluating the use of the yeast *Debaryomyces hansenii* as a biological control for postharvest strawberry quality found that fungal community diversity, composition, and structure were altered on treated fruit surfaces (Ferreira et al., 2024). At the phylum level, *Proteobacteria*, *Firmicutes*, *Bacteroidota*, and *Actinobacteria* were dominant across all groups. At the family level, *Pseudomonadaceae*, *Yersiniaceae*, and *Erwiniaceae* were the most abundant taxa (Supplementary Figures S3A,B), consistent with previous metagenomic studies on strawberries at various ripeness stages (Tenea and Reyes, 2024). At the genus level, all samples demonstrated a high diversity of microorganisms. Among these, *Pseudomonas* was the most abundant genus across most of the samples. However, a difference was observed in the sample PR3T1–0 from treatment T1, where *Serratia* was the dominant genus (Figure 6A). Similarly, *Serratia* was also prevalent in the samples PR3T3–0 and PR4T3–0 from treatment T3. This pattern suggests that the abundance of *Serratia* may be linked to specific factors associated with the experimental lot of fruits, potentially pointing to batch-related influence during the study, particularly the seasonal factors, temperature and humidity which may favor the increase of *Serratia.* Likewise, *Pseudomonas* was the most abundant genera across the groups on day 0 (21.72–48.32%), regardless of treatment. *Serratia* was notably prevalent in T1-0 (32.88%) and T3-0 (42.75%), while *Erwinia* was abundant in C1-0 (17.03%) (Figure 6B). By day 8, notable shifts in the proportions of genera were observed. *Pseudomonas* levels increased across all samples and among the groups, while *Serratia* significantly decreased, with the lowest levels recorded in C1-8 (1.15%) and T2-8 (2.78%), followed by T1-8 (6.66%) and T3-8 (6.51%). Similar reductions were noted for *Sphingomonas*. Conversely, *Pantoea* and *Erwinia*levels increased across all groups, regardless of treatment. Interestingly, *Achromobacter* (4.56%) was detected exclusively in T2-8, while *Clostridioides* (4.04%) appeared only in T3-8. These findings suggest that treatments influenced the microbiota both positively and negatively. For example, T1 and T2 treatments enriched beneficial genera such as *Weissella* (14.22%) and *Lactobacillus* (10.73%), while *Erwinia* and *Enterobacteriaceae* were depleted in T2-8 and T3-8, respectively. This pattern aligns with previous research on nectarines, where basil essential oil reduced *Monilinia* spp. epiphytically but allowed *Penicillium* to become more abundant (Remolif et al., 2024).

**Figure 7:**
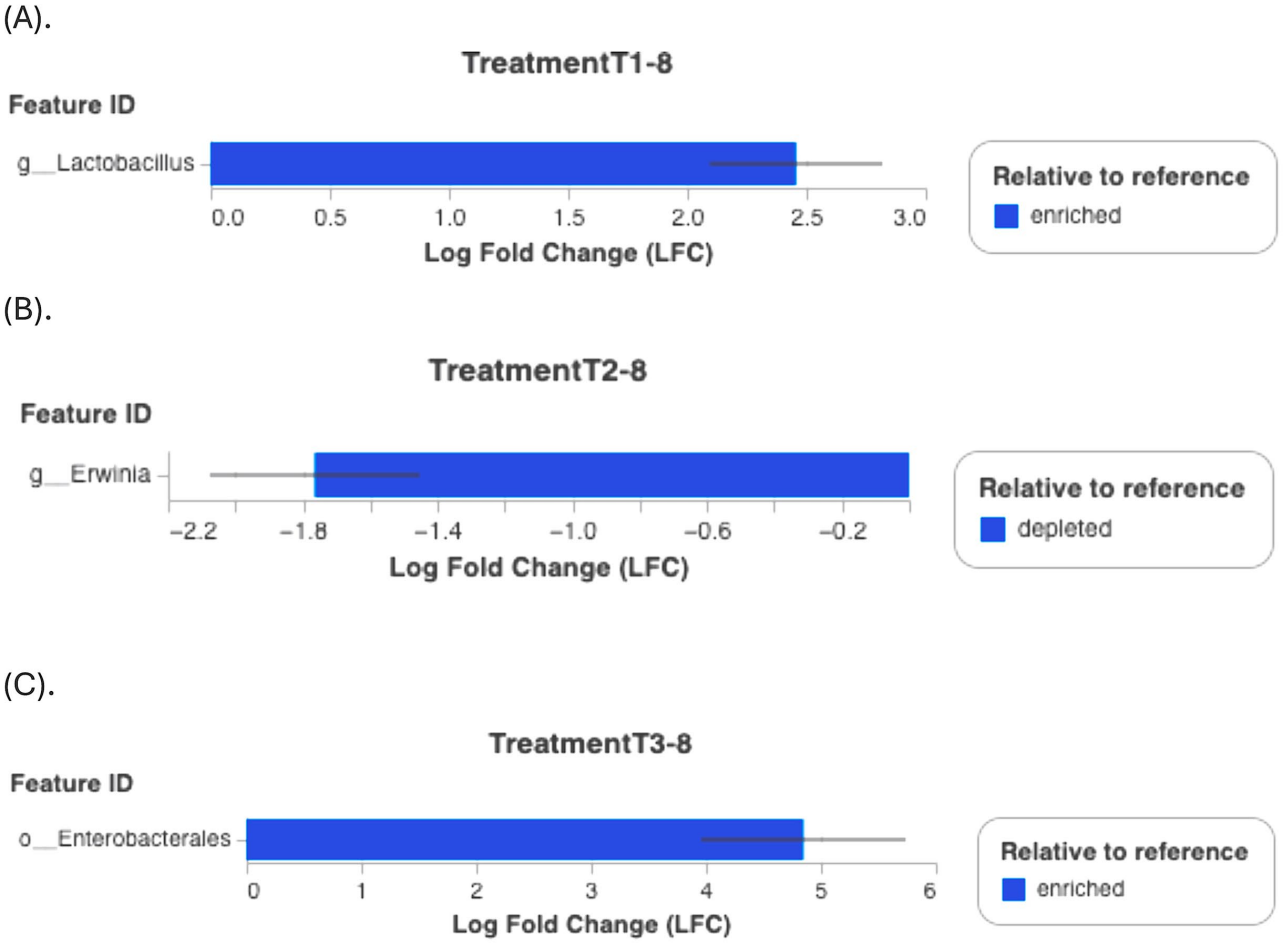
Differential abundance relative to the reference group (commercial disinfectant C1) using ANCOM-BC test. **(A)** T1-8; **(B)** T2-8; **(C)** T3-8. Y-axis (Feature ID): Lists the identifiers of the features (taxa) being compared. X-axis (Log Fold Change, LFC): Shows the LFC in abundance of each feature between the groups being compared. A positive value indicates that the feature is more enriched (more abundant) in the group of interest, while a negative value indicates that it is more depleted (less abundant). Error bars: Indicate variability or uncertainty in the Log Fold Change estimate.

Postharvest storage is known to substantially alter the microbial community on ready-to-eat fruits, with changes influenced by temperature and fungicide application (Umar et al., 2023). Although a lack of studies on the effects of antimicrobial agents on strawberry microbiota limits direct comparisons, the results demonstrate that genera proportions varied by group, time, and treatment. Differential abundance testing (ANCOM-BC) confirmed significant shifts, such as an increase in *Lactobacillus* abundance in T1-8 (*p* < 0.001, Figure 7A) compared to C1-8. T2-8 and T3-8 treatments also exhibited specific taxonomic changes, with *Enterobacteriaceae* becoming more abundant and *Erwinia* showing depletion (Figures 7B,C). Our findings suggest that PBFs can modify microbial diversity, potentially enhancing fruit preservation, nonetheless, the study focus on day 8 observations, which may only capture short-term effects of the treatments. Additionally, the increased presence of *Lactobacillus* suggests a potential role in shaping the postharvest microbial environment of strawberries. Through its natural antimicrobial activity, *Lactobacillus* may help extend shelf life and reduce microbial spoilage, highlighting its potential as a biocontrol agent in fruit preservation (Agriopoulou et al., 2020). Longer-term studies would be necessary to understand the sustained or delayed effects of the treatments on the microbiota. It’s unclear if the shifts observed on Day 8 would persist over a longer period or if they represent temporary changes. While certain genera may be associated with positive effects in other studies, their roles in strawberry microbiota or the specific context of the experiment may require more thorough investigation to determine their actual impact on strawberry health or disease resistance.

The raw sequence data from this study have been deposited in the National Center for Biotechnology Information under accession number PRJNA1199575 and BioProject SUB14937696 released on December 19, 2024^1^.

### 3.4 Principal component analysis (PCA) and correlation insights

**Figure 8:**
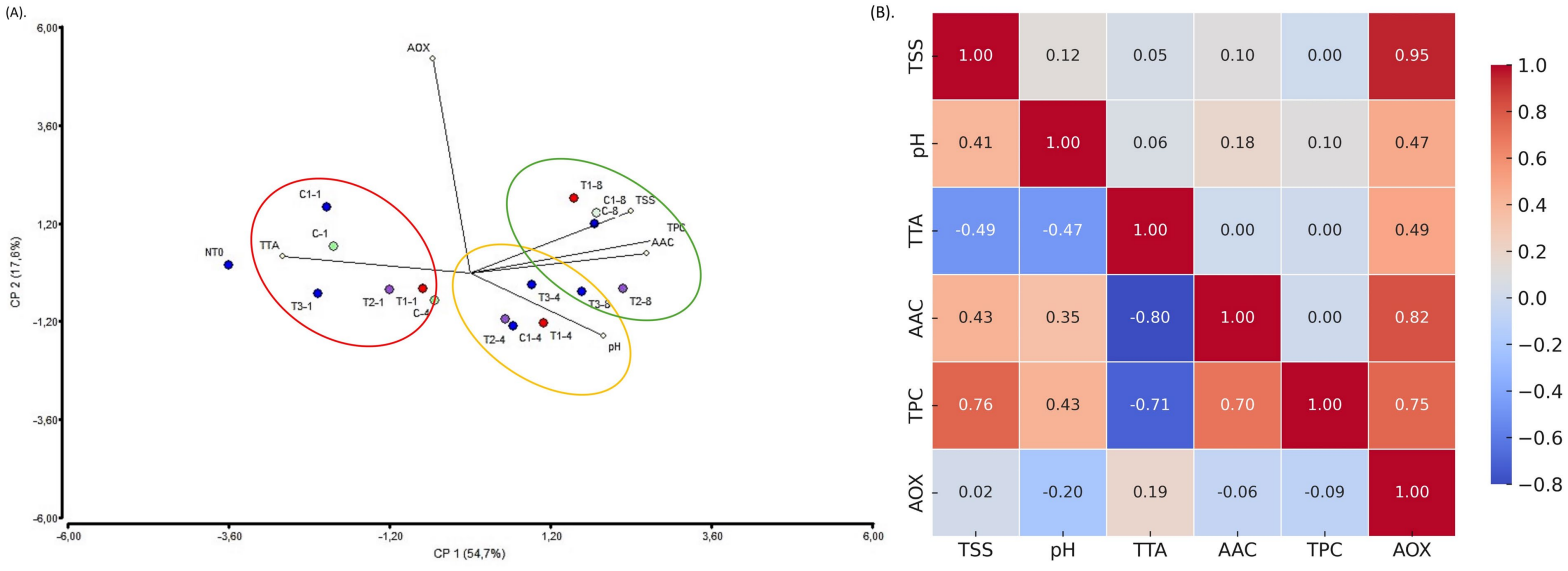
**(A)** Biplot PCA analysis of the six variables of treated and non-treated fruits during storage. The colors marked the close-related samples registered for each variable at day 1, 4, and 8 of storage. **(B)** Heatmap visualization of the correlation matrix for the variables evaluated in treated and non-treated strawberries. The color gradient represents the strength and direction of correlations, with red indicating strong positive correlations and blue indicating negative correlations. Legend: T1: (1 × MIC) PPGt21O + EPSCys2-2 (1:1, v/v); T2: (1 × MIC) PPGt21O; T3: (1 × MIC) EPSCys2-2; C: P4StpC1 untreated cell culture; C1, commercial disinfectant treated cells. TSS, total soluble solids; TAA, total titratable acidity; AOX, antioxidant capacity; TPC, total polyphenol content; AAC, ascorbic acid content.

To explore an eco-friendly and effective method of quality preservation, we treated strawberries with postbiotic formulations (PBFs) and evaluated their effects during refrigerated storage. PCA analysis of six key variables—pH, titratable acidity (TTA), total soluble solids (TSS), ascorbic acid content (AAC), total polyphenol content (TPC), and antioxidant capacity (AOX)—revealed a clear separation based on storage time rather than treatment (Figure 8A). Supplementary Table S5 showed the registered parameters values overtime. The first principal component (F1) accounted for 54.7% of the total variance, while the second component (F2) explained 17.6%. The results indicated that pH and acidity were higher during the early days of storage (days 1 and 4), whereas concentrations of ascorbic acid, polyphenols, and total soluble solids increased by day 8. Notably, the AOX vector formed a 90-degree angle with the other vectors, suggesting it operates independently or has a weak relationship with the other variables. Pearson correlation analysis highlighted the strongest correlations between TSS and AOX (0.93), AAC and AOX (0.82), and AOX and TPC (0.75) (Figure 8B). An inverse relationship was observed between TTA and AAC, with a correlation of −0.80. Other notable correlations included TPC and TSS (0.76) and TPC and TAA (−0.71). These findings align with earlier studies, such as the treatment of avocados with LAB-derived metabolites, where no significant quality changes were observed during room-temperature storage (Tenea et al., 2024b). Similarly, strawberries coated with chitosan (0.5%), guava leaf-based chitosan nanoparticles, or their combination showed negligible changes in TSS, TTA, and pH compared to untreated fruits during storage (Ali et al., 2022). Complementary analysis indicated that firmness varied over time. On day 1, all fruits exhibited a similar pattern, but from days 4 to 8, the recorded Newton values were lower for the T3 and control (C) samples, suggesting that they were softer compared to those treated with T1 and T2 (Supplementary Figure S4). The observed changes in firmness may be attributed to alterations in the activity of key softening enzymes, such as polygalacturonase, pectin methylesterase, and *β*-galactosidase, which play a crucial role in cell wall degradation (Moya-León et al., 2019). Additionally, microbial activity could contribute to textural modifications, either through direct enzymatic action or by influencing the fruits biochemical environment (Wang et al., 2020). Although it is currently difficult to establish a definitive firmness profile, the results are encouraging. Understanding the interplay between enzymatic processes and microbial dynamics is essential for elucidating the mechanisms underlying postharvest texture changes. According to a recent study, the temporal expression patterns of multiple cell wall-associated genes during the ripening and postharvest stages regulate differences in fruit firmness and shelf life among cultivated strawberries (Ren et al., 2023). Future studies with samples from multiple harvest seasons and a broader range of treatment conditions are needed to validate these findings.

## 4 Conclusion

This study highlights the significant antibacterial potential of postbiotic formulations (PBFs) derived from two native lactic acid bacteria in controlling *Serratia liquefaciens* isolated from strawberries. Specifically, a crosslink between precipitated peptide-protein extracts and exopolysaccharides effectively reduced bacterial growth both *in vitro* and when applied to the fruit surface. The PBFs induced structural alterations and cell wall damage in the bacteria, ultimately leading to cell death. Pre-treatment of ripe-stage strawberries with PBFs minimized microbial colonization on the fruit surface, reducing fruit damage while preserving quality attributes. These findings highlight the potential of the tested formulations to reduce bacterial contamination post-harvest, laying the groundwork for developing innovative fruit bio-protectors derived from LAB metabolites. Furthermore, 16S metagenomic analysis revealed both beneficial and adverse effects of the treatments on the fruit microbiota. By day eight of storage, the mean absolute abundance of the *Lactobacillus* genus was significantly higher (*p* < 0.001) in the treatment group (PPGt21O + EPSCys2-2) compared to the control disinfectant group (C1-8), as determined by the ANCOM-BC test. This suggests that the formulation may foster the growth of beneficial microorganisms, contributing to its overall antimicrobial effectiveness. The study revealed that the PBF coating effectively extended the shelf life of strawberries, maintained fruit quality, and slowed down the deterioration process, providing a sustainable and environmentally friendly approach to enhancing post-harvest properties. However, the study should be further expanded to include multiple strawberry varieties and different seasons to ensure more comprehensive and reliable findings. Besides, these preliminary study does not address the practical challenges of scaling up the production of the PBFs or their feasibility for commercial application, such as cost-effectiveness, regulatory compliance, or large-scale reproducibility. Future research should investigate the formulation’s applicability in different conditions such as varying temperatures, humidity levels, or handling practices.

## Data Availability

The raw sequence data from this study have been deposited in the National Center for Biotechnology Information under accession number PRJNA1199575.

## Footnotes

^1^ https://www.ncbi.nlm.nih.gov/sra/PRJNA1199575
