# Supplementary material for: Crosslinking bacterial postbiotics for microbial and quality control of strawberries postharvest: bacteriological and 16S amplicon metagenome evidence
Source: Front Microbiol. 2025 Mar 19;16:1570312. doi: 10.3389/fmicb.2025.1570312 (PMC11961906; doi:10.3389/fmicb.2025.1570312)

**Supplementary files**

**Crosslinking Bacterial Postbiotics for Microbial and Quality Control of Strawberries Postharvest: Bacteriological and 16S Amplicon Metagenome Evidence**

**Gabriela N. Tenea*, Pamela Reyes, Carlos Flores**

Biofood and Nutraceutics Research and Development Group, Faculty of Engineering in Agricultural and Environmental Sciences, Universidad Tecnica del Norte, Av. 17 de Julio s-21. Barrio El Olivo, Postcode: 100150, Ibarra, Ecuador

*Correspondence:

Corresponding author: Gabriela N. Tenea

gntenea@utn.edu.ec

**Table S1.** Description of PBFs composition and zone of inhibition (ZOI) towards *Serratia liquefaciens* P4StpC1. Legend: PPGt21O: postbiotics extract from *W. cibaria* strain UTNGt21O; EPSCys2-2: exopolysaccharide extract from *W. confusa* strain UTNCys2-2; MIC- minimum inhibitory concentration.

| **Code PBFs** | **Description (concentration/ postbiotic extract LAB strain code)** | **ZOI (mm)** |
| --- | --- | --- |
| T1 | (1 x MIC) PPGt21O + EPSCys2-2 (1:1, v/v) | 14.07 ± 0.05^A^ |
| T2 | (1 x MIC) PPGt21O | 14.33 ± 0.22^A^ |
| T3 | (1 x MIC) EPSCys2-2 | 9.01 ± 0.05^D^ |
| T4 | (1 x MIC) PPGt21O + EPSCys2-2 (1:3, v/v) | 11.33 ± 0.05^C^ |
| T5 | (1 x MIC) PPGt21O + EPSCys2-2 (3:1, v/v) | 12.33 ± 0.22^B^ |

Data are means ± standard error. Values with different letters are significantly different *P* < 0.05.

Table S2. Filtered raw data summary

| **Samples Description** | **Sample-ID** | **Input** | **Filtered** | **Percentage of input passed filter** | **Denoised** | **Merged** | **Percentage of input merged** | **Non-chimeric** | **Percentage of input non-chimeric** |
| --- | --- | --- | --- | --- | --- | --- | --- | --- | --- |
| C1: samples treated with commercial disinfectant at initial (0 day) and final (day 8) of storage | **P1-0** | 22976 | 16702 | 72.69 | 16585 | 16468 | 71.67 | 15542 | 67.64 |
|  | **PR1-0** | 92251 | 69175 | 74.99 | 69010 | 68748 | 74.52 | 67366 | 73.02 |
|  | **PR3-0** | 226809 | 178934 | 78.89 | 178379 | 176914 | 78 | 168274 | 74.19 |
|  | **PR4-0** | 230702 | 178134 | 77.21 | 177325 | 175254 | 75.97 | 163656 | 70.94 |
|  | **P1-8** | 19228 | 13626 | 70.87 | 13514 | 13360 | 69.48 | 12071 | 62.78 |
|  | **PR1-8** | 137927 | 106393 | 77.14 | 106123 | 105536 | 76.52 | 97013 | 70.34 |
|  | **PR3-8** | 208409 | 147678 | 70.86 | 147385 | 146981 | 70.53 | 143891 | 69.04 |
|  | **PR4-8** | 220162 | 163272 | 74.16 | 163009 | 162354 | 73.74 | 158285 | 71.89 |
| T1: samples treated with 1 x MIC (PPgt21O+ EPSCys2-2, 1:1 v/v) at initial (0 day) and final (day 8) of storage | **PT1-0** | 75124 | 54518 | 72.57 | 54283 | 53997 | 71.88 | 50981 | 67.86 |
|  | **PRT1-0** | 102003 | 74014 | 72.56 | 73716 | 73293 | 71.85 | 68360 | 67.02 |
|  | **PR3T1-0** | 193209 | 159143 | 82.37 | 158763 | 157458 | 81.5 | 156916 | 81.22 |
|  | **PR4T1-0** | 252189 | 197122 | 78.16 | 196520 | 195069 | 77.35 | 188215 | 74.63 |
|  | **PT1-8** | 47239 | 25388 | 53.74 | 25275 | 25106 | 53.15 | 24654 | 52.19 |
|  | **PRT1-8** | 206969 | 153369 | 74.1 | 152872 | 152008 | 73.44 | 146134 | 70.61 |
|  | **PR3T1-8** | 241609 | 185180 | 76.64 | 184953 | 183918 | 76.12 | 179665 | 74.36 |
|  | **PR4T1-8** | 267984 | 204081 | 76.15 | 203691 | 202701 | 75.64 | 182358 | 68.05 |
| T2: samples treated with 1 x MIC (PPgt21O) at initial (0 day) and final (day 8) of storage | **PT2-0** | 128669 | 96744 | 75.19 | 96396 | 95749 | 74.41 | 93122 | 72.37 |
|  | **PRT2-0** | 144516 | 106720 | 73.85 | 106231 | 105527 | 73.02 | 102324 | 70.8 |
|  | **PR3T2-0** | 159431 | 117570 | 73.74 | 116939 | 115611 | 72.51 | 113776 | 71.36 |
|  | **PR3T3-0** | 177905 | 141233 | 79.39 | 140872 | 140315 | 78.87 | 135205 | 76 |
|  | **PT2-8** | 73105 | 55275 | 75.61 | 54963 | 54497 | 74.55 | 44087 | 60.31 |
|  | **PRT2-8** | 72064 | 51846 | 71.94 | 51609 | 51309 | 71.2 | 40384 | 56.04 |
|  | **PR3T2-8** | 169160 | 130079 | 76.9 | 129874 | 129102 | 76.32 | 119017 | 70.36 |
|  | **PR3T3-8** | 207415 | 152670 | 73.61 | 152493 | 152092 | 73.33 | 146318 | 70.54 |
| T3: samples treated with 1 x MIC (EPSCys2-2) at initial (0 day) and final (day 8) of storage | **PT3-0** | 186480 | 133478 | 71.58 | 132737 | 131560 | 70.55 | 117010 | 62.75 |
|  | **PRT3-0** | 131275 | 97967 | 74.63 | 97586 | 96996 | 73.89 | 91811 | 69.94 |
|  | **PR4T2-0** | 182946 | 139453 | 76.23 | 138955 | 137828 | 75.34 | 131413 | 71.83 |
|  | **PR4T3-0** | 198569 | 153643 | 77.38 | 153044 | 150861 | 75.97 | 136891 | 68.94 |
|  | **PT3-8** | 74012 | 51847 | 70.05 | 51666 | 51193 | 69.17 | 48699 | 65.8 |
|  | **PRT3-8** | 109244 | 82347 | 75.38 | 82206 | 81866 | 74.94 | 77864 | 71.28 |
|  | **PR4T2-8** | 200362 | 152670 | 76.2 | 152493 | 152036 | 75.88 | 147426 | 73.58 |
|  | **PR4T3-8** | 233475 | 176650 | 75.66 | 176188 | 175205 | 75.04 | 162756 | 69.71 |

**Table S3**. Comparison of alpha-diversity in treated and no treated fruits based on Shannon index. C1: samples treated with commercial disinfectant at initial (0 day) and final (day 8) of storage; T1: samples treated with 1 x MIC (PPgt21O+ EPSCys2-2, 1:1 v/v) at initial (0 day) and final (day 8) of storage; T2: samples treated with 1 x MIC (PPgt21O) at initial (0 day) and final (day 8) of storage; T3: samples treated with 1 x MIC (EPSCys2-2) at initial (0 day) and final (day 8) of storage.

| **Group 1** | **Group 2** | **H** | **p-value** | **q-value** |
| --- | --- | --- | --- | --- |
| **C1-0 (n=4)** | C1-8 (n=4) | 0.33 | 0.56 | 0.83 |
| **C1-0 (n=4)** | T1-0 (n=4) | 0.33 | 0.56 | 0.83 |
| **C1-0 (n=4)** | T1-8 (n=4) | 0.08 | 0.77 | 0.83 |
| **C1-0 (n=4)** | T2-0 (n=4) | 0.33 | 0.56 | 0.83 |
| **C1-0 (n=4)** | T2-8 (n=4) | 0.00 | 1.00 | 1.00 |
| **C1-0 (n=4)** | T3-0 (n=4) | 0.08 | 0.77 | 0.83 |
| **C1-0 (n=4)** | T3-8 (n=4) | 0.08 | 0.77 | 0.83 |
| **C1-8 (n=4)** | T1-0 (n=4) | 0.33 | 0.56 | 0.83 |
| **C1-8 (n=4)** | T1-8 (n=4) | 1.33 | 0.25 | 0.83 |
| **C1-8 (n=4)** | T2-0 (n=4) | 0.33 | 0.56 | 0.83 |
| **C1-8 (n=4)** | T2-8 (n=4) | 1.33 | 0.25 | 0.83 |
| **C1-8 (n=4)** | T3-0 (n=4) | 0.33 | 0.56 | 0.83 |
| **C1-8 (n=4)** | T3-8 (n=4) | 2.08 | 0.15 | 0.83 |
| **T1-0 (n=4)** | T1-8 (n=4) | 0.08 | 0.77 | 0.83 |
| **T1-0 (n=4)** | T2-0 (n=4) | 0.08 | 0.77 | 0.83 |
| **T1-0 (n=4)** | T2-8 (n=4) | 0.00 | 1.00 | 1.00 |
| **T1-0 (n=4)** | T3-0 (n=4) | 0.75 | 0.39 | 0.83 |
| **T1-0 (n=4)** | T3-8 (n=4) | 0.33 | 0.56 | 0.83 |
| **T1-8 (n=4)** | T2-0 (n=4) | 0.33 | 0.56 | 0.83 |
| **T1-8 (n=4)** | T2-8 (n=4) | 0.33 | 0.56 | 0.83 |
| **T1-8 (n=4)** | T3-0 (n=4) | 0.08 | 0.77 | 0.83 |
| **T1-8 (n=4)** | T3-8 (n=4) | 0.08 | 0.77 | 0.83 |
| **T2-0 (n=4)** | T2-8 (n=4) | 0.33 | 0.56 | 0.83 |
| **T2-0 (n=4)** | T3-0 (n=4) | 0.75 | 0.39 | 0.83 |
| **T2-0 (n=4)** | T3-8 (n=4) | 0.75 | 0.39 | 0.83 |
| **T2-8 (n=4)** | T3-0 (n=4) | 0.33 | 0.56 | 0.83 |
| **T2-8 (n=4)** | T3-8 (n=4) | 0.33 | 0.56 | 0.83 |
| **T3-0 (n=4)** | T3-8 (n=4) | 0.08 | 0.77 | 0.83 |

*H value was compared with the critical values of the chi-square (χ²) distribution for k − 1 degrees of freedom, where k is the number of groups. If the calculated value of H is less than the critical chi-square value, there is insufficient evidence to reject the null hypothesis. This implies that there are no significant differences between the medians of the groups. If the calculated value of H is greater than the critical chi-square value, it means that there is sufficient evidence to reject the null hypothesis. This suggests that at least one of the groups has a median significantly different from the others.

**Table S4**. Beta-diversity metrics. The significance was determined through 999 Monte Carlo permutations; the values were considered significant when p < 0.05. A. Jaccard distance; B) (B) Bray–Curtis dissimilarity indices;(C) unweighted UniFrac distance, (D) weighted UniFrac distance. *Pseudo-F value determined according to Lattin et al. (2003).

(A).

| **Group 1** | **Group 2** | **Sample size** | **Permutations** | **pseudo-F** | **p-value** | **q-value** |
| --- | --- | --- | --- | --- | --- | --- |
| **C1-T0** | C1-T8 | 8 | 999 | 1.243 | 0.214 | 0.257 |
| **C1-T0** | T0 | 16 | 999 | 0.774 | 0.631 | 0.631 |
| **C1-T0** | T8 | 16 | 999 | 1.296 | 0.126 | 0.252 |
| **C1-T8** | T0 | 16 | 999 | 1.814 | 0.051 | 0.153 |
| **C1-T8** | T8 | 16 | 999 | 1.192 | 0.192 | 0.257 |
| **T0** | T8 | 24 | 999 | 1.812 | 0.022 | 0.132 |

(B).

| **Group 1** | **Group 2** | **Sample size** | **Permutations** | **pseudo-F** | **p-value** | **q-value** |
| --- | --- | --- | --- | --- | --- | --- |
| **C1-T0** | C1-T8 | 8 | 999 | 1.234 | 0.294 | 0.294 |
| **C1-T0** | T0 | 16 | 999 | 1.313 | 0.209 | 0.278 |
| **C1-T0** | T8 | 16 | 999 | 1.286 | 0.232 | 0.278 |
| **C1-T8** | T0 | 16 | 999 | 1.713 | 0.056 | 0.278 |
| **C1-T8** | T8 | 16 | 999 | 1.362 | 0.205 | 0.278 |
| **T0** | T8 | 24 | 999 | 1.301 | 0.220 | 0.278 |

(C).

| **Group 1** | **Group 2** | **Sample size** | **Permutations** | **pseudo-F** | **p-value** | **q-value** |
| --- | --- | --- | --- | --- | --- | --- |
| **C1-T0** | C1-T8 | 8 | 999 | 1.273 | 0.280 | 0.420 |
| **C1-T0** | T0 | 16 | 999 | 0.526 | 0.860 | 0.860 |
| **C1-T0** | T8 | 16 | 999 | 0.884 | 0.532 | 0.638 |
| **C1-T8** | T0 | 16 | 999 | 2.298 | 0.022 | 0.066 |
| **C1-T8** | T8 | 16 | 999 | 2.039 | 0.014 | 0.066 |
| **T0** | T8 | 24 | 999 | 1.851 | 0.060 | 0.120 |

(D).

| **Group 1** | **Group 2** | **Sample size** | **Permutations** | **pseudo-F** | **p-value** | **q-value** |
| --- | --- | --- | --- | --- | --- | --- |
| **C1-T0** | C1-T8 | 8 | 999 | 1.639 | 0.167 | 0.245 |
| **C1-T0** | T0 | 16 | 999 | 1.111 | 0.245 | 0.245 |
| **C1-T0** | T8 | 16 | 999 | 1.817 | 0.132 | 0.245 |
| **C1-T8** | T0 | 16 | 999 | 1.246 | 0.216 | 0.245 |
| **C1-T8** | T8 | 16 | 999 | 1.524 | 0.203 | 0.245 |
| **T0** | T8 | 24 | 999 | 2.051 | 0.131 | 0.245 |

**Table T5.** Physicochemical registered values for strawberries during storage (days 0, 1, 4 and 8).

| **Sample** | **Group- Time (day)** | **TSS** | **pH** | **TTA** | **AAC** | **TPC** | **AOX** |
| --- | --- | --- | --- | --- | --- | --- | --- |
| 1 | T1-0 | 8.2 | 3.61 | 0.18 | 321 | 263 | 5042 |
| 2 | T1-0 | 8.3 | 3.65 | 0.19 | 319 | 261 | 5043 |
| 3 | T1-0 | 8.5 | 3.58 | 0.19 | 319 | 262 | 5042 |
| 4 | T1-0 | 8.6 | 3.53 | 0.18 | 319 | 263 | 5043 |
| 5 | T2-0 | 8.2 | 3.61 | 0.18 | 321 | 263 | 5042 |
| 6 | T2-0 | 8.3 | 3.65 | 0.19 | 319 | 261 | 5043 |
| 7 | T2-0 | 8.5 | 3.58 | 0.19 | 319 | 262 | 5042 |
| 8 | T2-0 | 8.6 | 3.53 | 0.18 | 319 | 263 | 5043 |
| 9 | T3-0 | 8.2 | 3.61 | 0.18 | 321 | 263 | 5042 |
| 10 | T3-0 | 8.3 | 3.65 | 0.19 | 319 | 261 | 5043 |
| 11 | T3-0 | 8.5 | 3.58 | 0.19 | 319 | 262 | 5042 |
| 12 | T3-0 | 8.6 | 3.53 | 0.18 | 319 | 263 | 5043 |
| 13 | C1-0 | 8.2 | 3.61 | 0.18 | 321 | 263 | 5042 |
| 14 | C1-0 | 8.3 | 3.65 | 0.19 | 319 | 261 | 5043 |
| 15 | C1-0 | 8.5 | 3.58 | 0.19 | 319 | 262 | 5042 |
| 16 | C1-0 | 8.6 | 3.53 | 0.18 | 319 | 263 | 5043 |
| 17 | C-0 | 8.2 | 3.61 | 0.18 | 321 | 263 | 5042 |
| 18 | C-0 | 8.3 | 3.65 | 0.19 | 319 | 261 | 5043 |
| 19 | C-0 | 8.5 | 3.58 | 0.19 | 319 | 262 | 5042 |
| 20 | C-0 | 8.6 | 3.53 | 0.18 | 319 | 263 | 5043 |
| 21 | T1-1 | 8.2 | 3.59 | 0.15 | 348 | 284 | 5013 |
| 22 | T1-1 | 8.3 | 3.66 | 0.16 | 347 | 282 | 5012 |
| 23 | T1-1 | 8.5 | 3.59 | 0.21 | 348 | 283 | 5013 |
| 24 | T1-1 | 8.6 | 3.58 | 0.15 | 348 | 282 | 5013 |
| 25 | T2-1 | 8.2 | 3.64 | 0.19 | 388 | 262 | 5034 |
| 26 | T2-1 | 8.3 | 3.62 | 0.15 | 387 | 263 | 5035 |
| 27 | T2-1 | 8.5 | 3.61 | 0.20 | 389 | 264 | 5033 |
| 28 | T2-1 | 8.6 | 3.60 | 0.16 | 389 | 263 | 5034 |
| 29 | T3-1 | 8.2 | 3.67 | 0.17 | 397 | 282 | 5038 |
| 30 | T3-1 | 8.3 | 3.75 | 0.14 | 396 | 281 | 5040 |
| 31 | T3-1 | 8.5 | 3.57 | 0.18 | 397 | 281 | 5041 |
| 32 | T3-1 | 8.6 | 3.61 | 0.17 | 398 | 282 | 5040 |
| 33 | C1-1 | 8.2 | 3.57 | 0.18 | 362 | 276 | 5086 |
| 34 | C1-1 | 8.3 | 3.67 | 0.20 | 363 | 277 | 5085 |
| 35 | C1-1 | 8.5 | 3.61 | 0.15 | 363 | 277 | 5086 |
| 36 | C1-1 | 8.6 | 3.59 | 0.18 | 364 | 277 | 5085 |
| 37 | C-1 | 8.2 | 3.62 | 0.19 | 334 | 270 | 5063 |
| 38 | C-1 | 8.3 | 3.65 | 0.17 | 334 | 272 | 5062 |
| 39 | C-1 | 8.5 | 3.65 | 0.19 | 333 | 270 | 5064 |
| 40 | C-1 | 8.6 | 3.65 | 0.19 | 334 | 270 | 5063 |
| 41 | T1-4 | 10.8 | 3.63 | 0.15 | 356 | 286 | 5007 |
| 42 | T1-4 | 9.1 | 3.69 | 0.13 | 354 | 285 | 5008 |
| 43 | T1-4 | 9.1 | 3.67 | 0.17 | 356 | 287 | 5008 |
| 44 | T1-4 | 8.1 | 3.64 | 0.14 | 355 | 287 | 5010 |
| 45 | T2-4 | 9.6 | 3.64 | 0.15 | 440 | 286 | 5016 |
| 46 | T2-4 | 9.2 | 3.64 | 0.14 | 442 | 287 | 5017 |
| 47 | T2-4 | 9.5 | 3.61 | 0.16 | 440 | 286 | 5017 |
| 48 | T2-4 | 8.7 | 3.64 | 0.16 | 440 | 287 | 5018 |
| 49 | T3-4 | 9.5 | 3.64 | 0.12 | 428 | 283 | 5003 |
| 50 | T3-4 | 9.2 | 3.70 | 0.15 | 429 | 283 | 5002 |
| 51 | T3-4 | 8.8 | 3.61 | 0.18 | 427 | 285 | 5003 |
| 52 | T3-4 | 8.4 | 3.64 | 0.13 | 429 | 285 | 5004 |
| 53 | C1-4 | 9.7 | 3.64 | 0.13 | 408 | 288 | 5002 |
| 54 | C1-4 | 8.8 | 3.67 | 0.16 | 407 | 287 | 5001 |
| 55 | C1-4 | 8.6 | 3.61 | 0.15 | 407 | 288 | 5001 |
| 56 | C1-4 | 7.8 | 3.65 | 0.12 | 407 | 287 | 5000 |
| 57 | C-4 | 10.8 | 3.63 | 0.16 | 352 | 278 | 5012 |
| 58 | C-4 | 9.5 | 3.69 | 0.17 | 352 | 280 | 5012 |
| 59 | C-4 | 9.6 | 3.59 | 0.19 | 351 | 278 | 5013 |
| 60 | C-4 | 7.9 | 3.66 | 0.14 | 352 | 279 | 5012 |
| 61 | T1-8 | 10.7 | 3.67 | 0.14 | 422 | 289 | 5028 |
| 62 | T1-8 | 9.6 | 3.70 | 0.11 | 422 | 290 | 5027 |
| 63 | T1-8 | 10.1 | 3.65 | 0.16 | 422 | 290 | 5027 |
| 64 | T1-8 | 8.2 | 3.65 | 0.12 | 420 | 291 | 5026 |
| 65 | T2-8 | 10.5 | 3.61 | 0.14 | 451 | 294 | 5001 |
| 66 | T2-8 | 9.5 | 3.60 | 0.13 | 451 | 296 | 5002 |
| 67 | T2-8 | 8.9 | 3.6 | 0.16 | 453 | 294 | 5003 |
| 68 | T2-8 | 9.0 | 3.68 | 0.14 | 452 | 295 | 5002 |
| 69 | T3-8 | 9.8 | 3.70 | 0.11 | 440 | 286 | 5086 |
| 70 | T3-8 | 9.5 | 3.71 | 0.12 | 440 | 286 | 5090 |
| 71 | T3-8 | 9.1 | 3.59 | 0.17 | 440 | 285 | 5088 |
| 72 | T3-8 | 10.1 | 3.57 | 0.13 | 441 | 287 | 5087 |
| 73 | C1-8 | 9.8 | 3.60 | 0.12 | 427 | 301 | 5068 |
| 74 | C1-8 | 9.6 | 3.65 | 0.13 | 429 | 300 | 5069 |
| 75 | C1-8 | 9.7 | 3.65 | 0.15 | 429 | 301 | 5068 |
| 76 | C1-8 | 8.7 | 3.61 | 0.11 | 429 | 301 | 5067 |
| 77 | C-8 | 10.8 | 3.61 | 0.14 | 425 | 301 | 5050 |
| 78 | C-8 | 10.3 | 3.62 | 0.17 | 424 | 302 | 5051 |
| 79 | C-8 | 10.4 | 3.66 | 0.20 | 425 | 301 | 5050 |
| 80 | C-8 | 9.3 | 3.66 | 0.13 | 425 | 300 | 5051 |

C-0, C-1, C-4, C-8: samples washed with distillate water at day 0, 1, 4 and 8 of storage; C1-0, C1-1, C1-4, C-1-8: samples treated with commercial disinfectant at day 0, 1, 4 and 8 of storage; T1-0, T1-1, T1-4, T1-8: samples treated with 1 x MIC (PPgt21O+ EPSCys2-2, 1:1 v/v) day 0, 1, 4 and 8 of storage; T2-0, T2-1, T2-4, T2-8: samples treated with 1 x MIC (PPgt21O) at day 0, 1, 4 and 8 of storage; T3-0, T3-1, T3-4, T3-8: samples treated with 1 x MIC (EPSCys2-2) at day 0, 1, 4 and 8 of storage. TSS: total soluble solids, TAA: total titratable acidity, AOX: antioxidant capacity, TPC: total polyphenol content; AAC: ascorbic acid content.

**Figure S1.** Phenotypic aspect of strawberries at the initial point of experimentation (A). Resume of the subjective attributes (color, aspect) of strawberries upon the treatment with PBFs at 8 days of storage (B). Legend: C-control, water washed fruits; C1- fruits treated with commercial disinfectant; T1: fruits treated with PPGt21O + EPSCys2-2; T2: fruits treated with PPGt21O; T3: fruits treated with EPSCys2-2.

**Figure S2**. Refraction curves illustration.


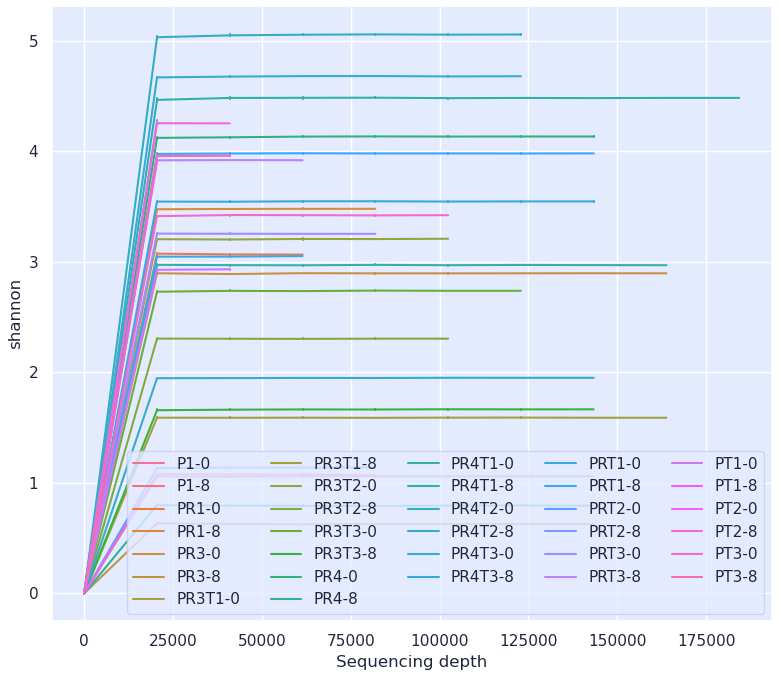


**Figure S3**. Relative abundance of different bacteria families across the samples (A) and among the groups (B) identified in strawberries upon the treatments at initial (day 0) and final (day 8) storage. This bar chart shows the relative abundance of the top 10 classification results within each taxonomic level. The "Other" category in this sum of all classifications with less than 0.15 % abundance. Legend: C1-0, C1-8: fruits treated with commercial disinfectant, time initial (0) and final (8); T1-0, T1-8: treatment with 1 x MIC (PPGt21O+EPSCys2-2, 1:1 v/v) time initial (0) and final (8); T2-0; T2-8: treatment with PPGt21O (1 x MIC) time initial (0) and final (8); T3-0, T3-8: treatment with EPSCys-2-2 (1 x MIC) time initial (0) and final (8). The samples ID are detailed in Table S2.

(A).


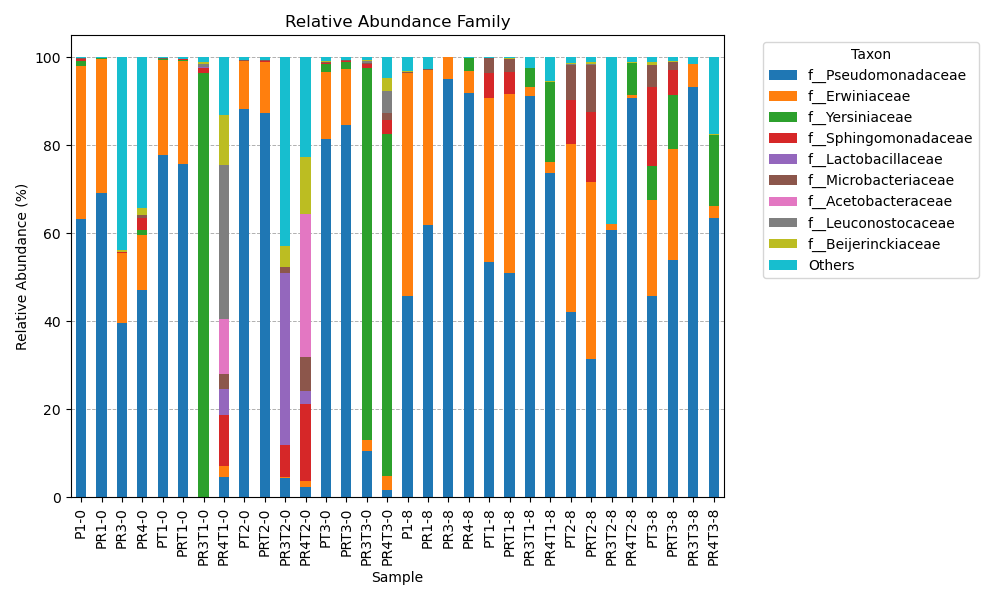


(B).


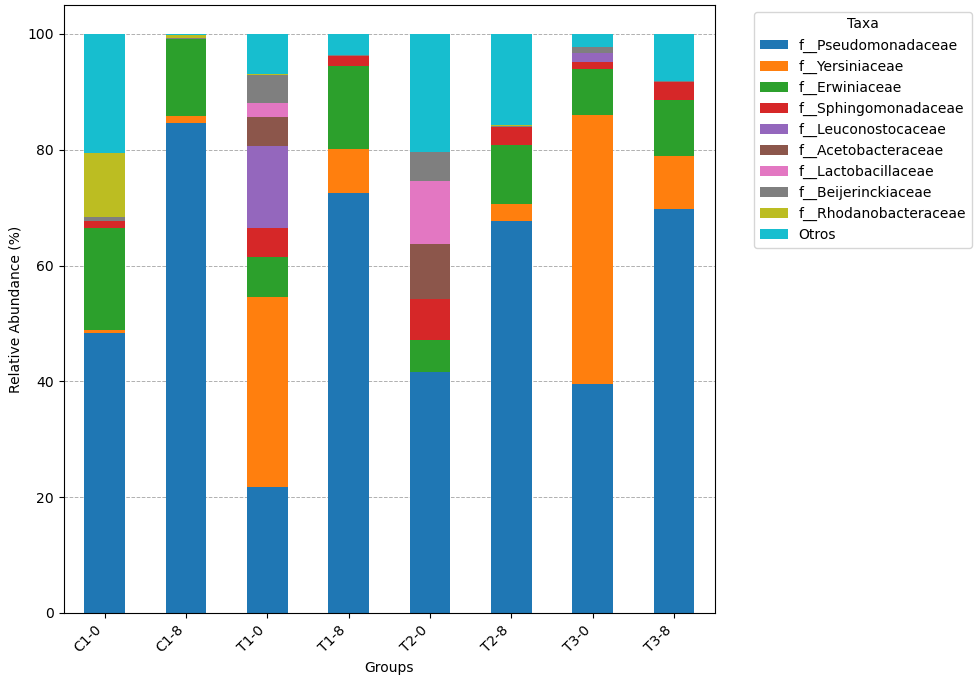


**Figure S4.** Variation of Newtons values of treated and no treated strawberries during storage. Legend: Legend: C-control, water washed fruits; C1- fruits treated with commercial disinfectant; T1: fruits treated with PPGt21O + EPSCys2-2; T2: fruits treated with PPGt21O; T3: fruits treated with EPSCys2-2.


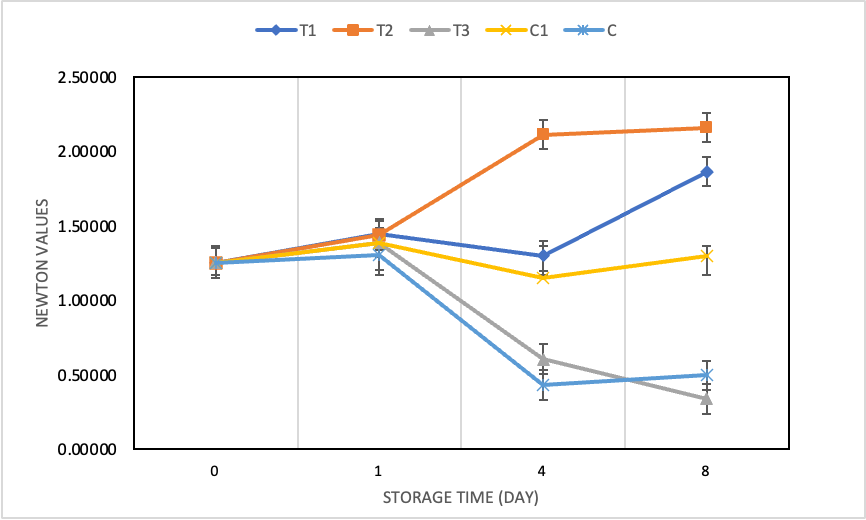

Supplement: Supplementary file 1 [file Data_Sheet_1.docx]
